# The accuracy of self-reported height, weight and BMI in a sample of emerging adult college students across California: an observational study

**DOI:** 10.1186/s12874-025-02745-5

**Published:** 2026-01-12

**Authors:** Isabella U. Yalif, Lindsay T. Hoyt, Lucia Calderón, Tatyana Bidopia, Natasha L. Burke, Benjamin W. Chaffee, Ryan Gamba, Serge Atherwood, Jiwoon Bae, Alison K. Cohen

**Affiliations:** 1 Department of Economics, Vanderbilt University, 2301 Vanderbilt Pl, Nashville, TN 37235 USA; 2 Department of Psychology, Fordham University, 441 E Fordham Rd, Bronx, New York, NY 10458 USA; 3 School of Medicine, Department of Epidemiology & Biostatistics, University of California San Francisco, 550 16Th Street, 2Nd Floor, San Francisco, CA 94158 USA; 4 School of Dentistry, Division of Oral Epidemiology and Dental Public Health, University of California San Francisco, 95 Kirkham Street Box 1361, San Francisco, CA 94066 USA; 5 Department of Public Health, California State University, East Bay, 25800 Carlos Bee Boulevard, Hayward, CA 94542 USA

**Keywords:** Emerging adults, Body mass index, Anthropometrics, Height, Weight, USA, College students, Self-report, Overweight

## Abstract

**Background:**

Self-reported height and weight are pragmatic, lower-cost alternatives for objective measurements but are potentially less accurate. This study examines the accuracy of self-reported measurements in a population of emerging adults.

**Methods:**

Participants were 603 emerging adult college students aged 18 to 24 who attend public Hispanic-Serving Institutions of higher education in California, USA. The population was heterogeneous by race/ethnicity, gender, socioeconomic position, and body size. Participants self-reported height and weight; height and weight were then objectively measured during an in-person visit.

**Results:**

Relative to objective measurements, participants, on average, overreported their height by 1.53 cm and underreported their weight by 0.77 kg, leading to an average body mass index (BMI) underestimation of 0.73 kg/m^2^. The discrepancy between self-report and objective measures significantly differed by gender, race/ethnicity, and weight but not by age, sexual orientation, household poverty status, or disordered eating behaviors. Overall, 23.8% of participants with overweight or obesity would be assigned to a lower-weight BMI category based on their self-reported measurements (including 16.2% to normal weight).

**Conclusions:**

In this sample of emerging adult college students, we found modest but statistically significant inaccuracies in self-reported height and weight that resulted in some misclassification of BMI-based weight status categories. Expressing self-reported BMI as a numerical value instead of categorically may be one approach to minimize bias. These results may inform bias-correction approaches in future emerging adult studies that lack the resources for in-person objective measures.

**Supplementary Information:**

The online version contains supplementary material available at 10.1186/s12874-025-02745-5.

## Background

Self-reported height and weight offer researchers an accessible and affordable way to obtain measurements, especially for highly mobile emerging adult (aged 18–24 years old) populations [1–3] where resource, geographical, accessibility, and/or other constraints may make it infeasible to directly measure participants’ height and weight [4, 5]. However, self-reported measurements may be less accurate and less precise, which could introduce statistical bias and potentially lead to misclassified body mass index (BMI) categories [5].

Many studies have examined the validity of self-reported measures across the lifecourse [1, 4–12]. While nearly all agree on the general validity of self-reported measures as proxies for objective anthropometric measurements, people, and emerging adults specifically, tend to overreport their height and underreport their weight [3, 9, 13]. This misreporting leads to an underreported BMI and, subsequently, misclassification of BMI category [14]. For example, in a nationally representative sample of US emerging adults, participants overreported height by 1.0 to 1.7 cm, and underreported weight by 0.6 to 1.7 kg [9]. Over 40% of emerging adults weigh themselves less than once per month, which may explain the weight inaccuracy [15].

College is a unique context for health promotion [16], and emerging adulthood is a key developmental window for promoting health– including healthy weight– across the lifecourse [17]. While previous studies have compared self-reported and objective measurements in emerging adult college students [1, 2, 9, 13, 18–21], most samples were of predominantly white participants [14, 19], and few studies analyzed differences in reporting accuracy by race/ethnicity or other sociodemographic characteristics beyond gender. Men tended to overreport their height but more accurately report their weight, whereas women tended to underreport their weight and more accurately report their height [1, 2, 18]. No studies, to the best of our knowledge, considered transgender and gender-diverse (TGD) participants. Additionally, for emerging adults, one study found that Black men, Black women, Hispanic women, and Native American men all provided height and weight estimates which resulted in more accurate self-reported BMIs than their white gender-matched peers of similar weights [10]. The limited other research on race/ethnicity-based differences in misreporting is mixed [2, 9]. Studies that considered participants’ weight categories found that people with overweight and obesity underreport their weight significantly more than peers with normal weight or underweight [18, 22].

The aim of this study was to examine how accurately emerging adult college students self-report their height and weight compared to their directly-assessed objective measurements.

## Methods

### Study design

This cross-sectional study analyzed participants in the 3E (Economic and Educational Contributors to Emerging Adults’ Cardiometabolic and Oral Health) Study. The 3E Study is a longitudinal cohort study that follows emerging adult college students attending public institutions of higher education over three years to study factors affecting cardiometabolic and oral health. Data for this paper only included information collected in the first survey and health visit for each participant that completed these items between September 2023 and May 2025.

Participants were recruited via emails to all eligible students, per lists generated by each university’s institutional research offices. We also recruited participants through on-campus events and tabling, Instagram posts and targeted Instagram advertisements, the 3E study website, presentations in university classes by members of the study team at each school, and word of mouth (including referral bonuses for participants who recruited peers to enroll). Participants received a gift card for completing the survey and health visit.

### Study sample

The 3E Study recruits all emerging adults aged 18 to 24 who are first-years, sophomores, or new transfer students without prior four-year college experience enrolled at two public Hispanic-Serving Institutions of higher education in California: the University of California, Riverside (UCR) and California State University, East Bay (CSUEB). While many emerging adults are quite mobile, by recruiting emerging adult college students, we collected data on a less mobile subsect of this population.

By May 29, 2025, 752 participants completed both the baseline survey and health visit. We excluded 149 (19.8%) participants from the analytic sample: 119 who did not self-report height or weight in the baseline survey; 14 who did not have height and/or weight measured in the health visit; 10 who had implausibly large differences between self-reported and measured height or weight (defined as 4 standard deviations above or below the mean difference) that were assumed to be data entry errors (e.g., two individuals whose measured height was 888 cm when their self-reported height was 160 cm, and one participant whose measured weight was implausibly listed as 15.7 kg); and 6 who participated in weigh-ins for college athletics or ROTC (a college-based training program for the US armed forces), as they may be more familiar with their true weight than others. We continued the analysis with the remaining 603 participants’ data. In comparison to the original sample, the analytical sample had a slightly higher percentage of participants above the poverty level, fewer participants missing income information, and a higher percentage of participants with disordered eating behaviors (Additional File 1).

The study was approved by the Fordham University Institutional Review Board. All participants in the study gave informed consent to participate.

### Measures

#### Weight and height

Participants completed both an online survey and an in-person set of objective measurements. In the survey, participants self-reported their height and weight to the nearest inch (height) and pound (weight), which we then converted to centimeters and kilograms, respectively. After completing the survey, participants attended a health visit conducted by trained research assistants. The median amount of time between a participant completing their survey and health visit was 6 days (interquartile range = 9 days; mean = 16.6 days, *SD* = 48.2 days). The large difference between median and mean lag from survey to health visit indicates that a few observations with large delays largely drove the high mean. We measured participant weight in minimal clothing (e.g., without shoes or heavy outer layers; all participants were in California, where there are not substantial seasonal differences in apparel) using a Tanita BC-1000 plus Total Body Composition Analyzer. (Research assistants explained to participants that the scale would only work if participants were barefoot; if participants did not want to remove their socks and shoes, an alternate scale (Oxiline Scale X Pro) was provided. However, it was highly uncommon for participants to decline to remove their socks and shoes.) Participant height was measured in triplicate without shoes and a loosened hair accessory (such as a hair elastic), if possible, using a fixed Health-O-Meter PORTROD Wall Mount Height Rod stadiometer. The three height measurements were then averaged. Weight was measured to the nearest 0.1 kg and height to the nearest 0.1 cm.

#### Body Mass Index (BMI)

BMI was computed using the formula [weight (kg)/height squared (m^2^)]. Self-reported BMI was calculated using self-reported weight and height. Objectively-measured BMI was calculated using the measured weight and height. We used BMI to classify participants into a weight group following the standard international adult BMI ranges (underweight: < 18.5 kg/m^2^, normal weight: 18.5 kg/m^2^ to < 25 kg/m^2^, overweight: 25 kg/m^2^ to < 30 kg/m^2^, obese: > 30 kg/m^2^) [23]. We note that BMI is widely acknowledged as an imperfect measure of nutritional status, body fat, and mortality risk [24].

#### Covariates

Participants self-reported gender (cisgender man, cisgender woman, trans man, trans woman, genderqueer/gender non-conforming, non-binary, different identity, not sure, prefer not to say), sex assigned at birth (male, female, intersex), sexual orientation (heterosexual or straight, bisexual, asexual, queer, pansexual, questioning, another sexual identity, don’t know, prefer not to say), race/ethnicity (open-ended response which was later coded by trained research assistants to white, Hispanic/Latino/Latine, Black or African American, Asian, American Indian or Alaskan Native, Middle Eastern or North African, Native Hawaiian or Pacific Islander, multiracial, prefer not to say), and household poverty level, based on self-reported annual total household income (from “less than $10,000” to “$150,000 and over,” starting with $4,999 increments up to $24,999 increments) and the number of people the household income supports. Household income was separated into *at or below poverty level*, > *poverty level, and missing information* (for those who did not report household income and/or the number of people the income supported). We used the BASE-10 to measure disordered eating behaviors [25]. Scores on the BASE-10 were summed to produce a total score. Individuals who scored eight or higher on the BASE-10 were classified as having clinically significant disordered eating.

For multiple variables, categories with fewer than 20 participants were collapsed for reporting and analysis due to small samples. For gender, trans man, trans woman, genderqueer/gender non-conforming, non-binary, and different identity were compiled into *trans/genderqueer/nonbinary* and not sure and prefer not to say were combined into *don’t know/prefer not to say*. For sexual orientation, gay, lesbian, bisexual, pansexual, and queer were grouped into *gay, lesbian, bisexual, pansexual, queer*, other sexual orientations were grouped into *another sexual orientation*, and don’t know and prefer not to say were not included due to small sample size. For race/ethnicity Black or African American, American Indian or Alaskan Native, and Middle Eastern or North African were condensed into *other race/ethnicity*. Asian and Pacific Islander were combined into AAPI (Asian/Asian American and Pacific Islander).

### Statistical analysis

Data analyses were conducted in Stata version 17. We calculated descriptive statistics (e.g., means and corresponding 95% CIs) for self-reported and objectively-measured height, weight, and BMI as well as the magnitude of misreporting (participant self-reported height/weight/BMI—objectively-measured height/weight/BMI) for the sample overall and stratified by participant characteristics. For each gender category, we calculated pairwise correlations by race/ethnicity. Bland–Altman plots were also constructed on height/weight/BMI reporting accuracy of the sample. Bland–Altman plots are a widely used method of evaluating the agreement between two methods, and have been used in other studies examining self-reported and objective measurements [26].

We conducted linear regressions to assess bivariate associations between participant characteristics and misreporting of height, weight, and BMI. We also calculated the percentages of participants whose BMI weight group would be misclassified based on self-reported height and weight, overall and for each weight group.

We conducted two sensitivity analyses. In order to compare the robustness of our results across the two universities, we stratified the regression models by research site. Next, to test robustness to potential lags between completing the survey (self-report) and the health visit (objective) measures, we stratified results by participants who had less than a week lag and those who had a week or more of a lag.

Because stratification by participant characteristics resulted in small subgroups for some variables, we did not report findings for subgroups of fewer than 10 participants to maintain participant confidentiality.

## Results

### Main analyses

The sample analyzed was varied by gender and race/ethnicity (Table 1). The sample was 55.1% cisgender women (*n* = 332) and 6.1% TGD (*n* = 37). Most of the sample was Hispanic/Latine (36.2%, *n* = 218) or AAPI (35.0%, *n* = 211), while the next largest subgroups, multiracial and white participants, made up 9.0% (*n* = 54) and 8.1% (*n* = 49) of the sample, respectively. The multiracial category consisted of many different combinations; the most common were white and AAPI (25.9%) and white and Hispanic/Latine (24.1%).

**Table 1:** Participant demographics and measurements

		**Mean (95% CI)**								
	**Analytical Sample Frequency (%)**	**Measured Height (cm)**	**Self-Reported Height (cm)**	**Difference in Height Values (Self-reported—Measured)**	**Measured Weight (kg)**	**Self-Reported Weight (kg)**	**Difference in Weight Values (Self-reported—Measured)**	**Measured BMI (kg/m**^**2**^**)**	**Self-Reported BMI (kg/m**^**2**^**)**	**Difference in BMI Values (Self-reported—Measured)**
Overall sample	603 (100.0)	164.56 (163.79, 165.33)	166.09 (165.26, 166.91)	1.53 (1.29, 1.77)	66.68 (65.34, 68.02)	65.94 (64.64, 67.25)	−0.77 (−1.03, −0.51)	24.54 (24.12, 24.96)	23.81 (23.41, 24.21)	−0.73 (−0.85, −0.61)
Gender										
Cisgender man	194 (32.2)	173.80 (172.83, 174.77)	176.26 (175.23, 177.28)	2.45 (1.92, 2.98)	76.85 (74.19, 79.52)	76.21 (73.67, 78.75)	−0.68 (−1.18, −0.18)	25.43 (24.60, 26.26)	24.48 (23.73, 25.24)	−0.95 (−1.18, −0.72)
Cisgender woman	332 (55.1)	159.54 (158.8, 160.24)	160.57 (159.86, 161.29)	1.04 (0.75, 1.33)	61.72 (60.28, 63.16)	60.82 (59.43, 62.20)	−0.90 (−1.22, −0.58)	24.25 (23.70, 24.79)	23.59 (23.07, 24.12)	−0.65 (−0.80, −0.50)
Trans/genderqueer/nonbinary	37 (6.1)	161.61 (158.76, 164.46)	162.97 (159.84, 166.11)	1.36 (0.80, 1.93)	62.12 (57.84, 66.40)	61.76 (57.58, 65.95)	−0.36 (−1.27, 0.55)	23.87 (22.15, 25.59)	23.38 (21.67, 25.08)	−0.49 (−0.87, −0.11)
Don’t know/prefer not to say	40 (6.6)	164.12 (160.64, 167.60)	165.42 (161.74, 169.09)	1.30 (0.64, 1.96)	63.06 (58.74, 67.38)	(62.58 (58.10, 67.05)	−0.49, (−1.69, 0.71)	23.29 (22.06, 24.51)	22.75 (21.50, 23.99)	−0.55 (−1.01, −0.07)
Sexual orientation										
Heterosexual or straight	412 (68.3)	165.33 (164.40, 166.26)	167.07 (166.08, 168.06)	1.74 (1.42, 2.06)	66.96 (65.41, 68.51)	66.30 (64.80, 67.80)	−0.70 (−1.00, −0.39)	24.43 (23.94, 24.91)	23.66 (23.22, 24.11)	−0.76 (−0.91, −0.62)
Gay, Lesbian, Bisexual, Pansexual, Queer	136 (22.6)	162.95 (161.30, 164.60)	164.06 (162.26, 165.87)	1.11 (0.71, 1.52)	68.84 (65.48, 72.21)	67.78 (64.47, 71.08)	−1.06 (−1.59, −0.54)	25.79 (24.70, 26.89)	25.05 (24.01, 26.09)	−0.74 (−0.98, −0.51)
Another sexual orientation	32 (5.3)	161.68 (158.70, 164.67)	162.84 (159.95, 165.73)	1.16 (0.32, 1.99)	56.93 (53.2, 60.66)	56.93 (53.05, 60.82)	0.00 (−1.17, 1.17)	21.72 (20.55, 22.89)	21.43 (20.16 (22.69)	−0.29 (−0.75, 0.16)
Don’t know/prefer not to say	23 (3.8)	164.22 (160.17, 168.27)	164.99 (160.81, 169.17)	0.77 (−0.09, 1.63)	62.57 (57.32, 67.82)	61.21 (56.22, 66.21)	−1.36 (−3.26, 0.55)	23.08 (21.74, 24.42)	22.41 (21.06, 23.76)	−0.67 (−1.25, −0.09)
Race/ethnicity										
White	49 (8.1)	168.55 (165.74, 171.36)	170.00 (167.01, 172.99)	1.45 (0.49, 2.40)	67.98 (64.10, 71.86)	68.23 (64.23, 72.23)	0.25 (−0.52, 1.02)	24.01 (22.58, 25.44)	23.66 (22.27, 25.04)	−0.35 (−0.74, 0.04)
Hispanic/Latine	218 (36.2)	162.94 (161.73, 164.15)	164.78 (163.49, 166.06)	1.83 (1.43, 2.24)	70.12 (67.64, 72.60)	59.11 (66.73, 71.50)	−1.01 (−1.46, −0.56)	26.31 (35.51, 27.11)	25.35 (24.61, 26.10)	−0.96 (−1.17, −0.74)
AAPI	211 (35.0)	163.85 (162.65, 165.05)	165.13 (163.81, 166.46)	1.28 (0.82, 1.75)	62.23 (60.29, 64.16)	61.38 (59.52, 63.23)	−0.85 (−1.24, −0.47)	23.02 (22.48, 23.57)	22.36 (21.87, 22.86)	−0.66 (−0.84, −0.48)
Other race/ethnicity	34 (5.6)	167.30 (163.27, 171.33)	169.21 (164.82, 173.59)	1.91 (0.95, 2.88)	69.71 (63.19, 76.24)	68.92 (62.65, 75.20)	−0.79 (−1.79, −0.21)	24.77 (22.90, 26.64)	23.94 (22.23, 25.65)	−0.82 (−1.21, −0.43)
Multiracial	54 (9.0)	167.50 (164.46, 170.54)	168.89 (165.63, 172.15)	1.38 (0.89, 1.88)	69.07 (64.15, 73.99)	68.84 (63.78, 73.90)	−0.23 (−1.27, 0.80)	24.56 (23.00, 26.11)	24.08 (22.50, 25.67)	−0.47 (−0.83, −0.11)
Don’t know/prefer not to say	37 (6.1)	165.98 (162.67, 169.29)	167.13 (163.82, 170.43)	1.14 (0.44, 1.84)	63.77 (59.33, 68.22)	63.35 (59.18, 67.51)	−1.00 (−2.27, 0.27)	23.22 (21.96, 24.48)	22.64 (21.36, 23.93)	−0.57 (−1.06, −0.09)
Poverty status										
At or below poverty level	89 (14.7)	162.62 (160.53, 164.71)	164.04 (161.83, 166.26)	1.43 (0.70, 2.15)	67.36 (63.68, 71.04)	66.21 (62.63, 69.79)	−1.15 (−1.79, −0.51)	25.41 (24.22, 26.60)	24.58 (23.42, 25.73)	−0.83 (−1.13, −0.53)
> Poverty level	118 (19.6)	164.34 (162.51, 166.17)	165.93 (164.00, 167.85)	1.58 (1.16, 2.01)	68.25 (64.81, 71.69)	67.70 (64.38, 71.03)	−0.68 (−1.24, −0.12)	25.10 (24.08, 26.12)	24.36 (23.43, 25.29)	−0.74 (−0.98, −0.50)
Missing income information	254 (42.1)	165.56 (164.41, 166.71)	167.13 (165.85, 168.41)	1.57 (1.13, 2.01)	66.13 (64.17, 68.09)	65.43 (63.54, 67.32)	−0.70 (−1.11, −0.29)	24.06 (23.45, 24.68)	23.36 (22.79, 23.93)	−0.70 (−0.89, −0.51)
[Missing value]	142 (23.5)	164.16 (162.59, 165.72)	165.64 (164.04, 167.24)	1.48 (1.11, 1.85)	65.95 (63.26, 68.65)	65.23 (62.58, 67.89)	−0.72 (−1.25, −0.19)	24.38 (23.50, 25.26)	23.68 (22.84, 24.52)	−0.71 (−0.94, −0.47)
Weight status (Calculated through measured BMI)										
Underweight (BMI < 18.5)	45 (7.5)	164.41 (161.64, 167.17)	165.88 (163.02, 168.74)	1.47 (0.83, 2.11)	47.33 (45.72, 48.91)	47.99 (46.28, 49.69)	0.66 (0.10, 1.22)	17.48 (17.22, 17.75)	17.41 (17.11, 17.71)	−0.07 (−0.28, 0.13)
Normal weight	336 (55.7)	164.65 (163.61, 165.69)	166.00 (164.91, 167.09)	1.34 (1.05, 1.63)	59.69 (58.73, 60.64)	59.29 (58.32, 60.28)	−0.39 (−0.68, −0.10)	21.93 (21.73, 22.12)	21.44 (21.24, 21.65)	−0.48 (−0.60, −0.36)
Overweight	140 (23.2)	164.21 (162.66, 165.75)	165.59 (163.95, 167.23)	1.38 (0.88, 1.89)	72.95 (71.50, 74.41)	71.73 (70.19, 73.27)	−1.31 (−1.87, −0.74)	27.01 (26.78, 27.24)	26.10 (25.80, 26.41)	−0.91 (−1.15, −0.67)
Obese (BMI ≥ 30)	82 (13.6)	164.85 (162.60, 167.11)	167.43 (164.79, 170.07)	2.58 (1.60, 3.56)	95.35 (91.53, 99.16)	93.14 (89.34, 96.95)	−2.20 (−3.19, −1.21)	34.90 (34.01, 35.79)	33.11 (32.16, 34.06)	−1.79 (−2.27, −1.30)
Disordered eating										
No	367 (60.9)	162.93 (162.03, 163.84)	164.33 (163.37, 165.30)	1.40 (1.12, 1.68)	63.96 (62.38, 65.55)	63.23 (61.70, 64.76)	−0.79 (−1.10, −0.48)	24.03 (23.51, 24.55)	23.34 (22.86, 23.83)	−0.69 (−0.83, −0.55)
Yes	209 (34.7)	167.00 (165.64, 168.37)	168.88 (167.40, 170.37)	1.88 (1.38, 2.38)	71.16 (68.69, 73.63)	70.40 (67.98, 72.82)	−0.76 (−1.21, −0.31)	25.46 (24.68, 26.25)	24.61 (23.88, 25.34)	−0.85 (−1.07, −0.64)
[Missing value]	27 (4.5)	167.67 (163.08, 172.28)	168.30 (163.56, 173.03)	0.62 (0.04, 1.20)	68.88 (62.78, 74.99)	68.36 (62.40, 74.32)	−0.52 (−2.32, 1.28)	24.32 (22.65, 25.98)	24.02 (22.31, 25.74)	−0.29 (−0.91, 0.32)

On average, in comparison to objective measurements, participants statistically significantly overreported their height by 1.53 cm (95% Confidence Interval (CI): 1.29, 1.77) and statistically significantly underreported their weight by 0.77 kg (95%CI: −1.03, −0.51), which led to self-reported BMI being statistically significantly underestimated (average error: −0.73 kg/m^2^, 95%CI: −0.85, −0.61). Self-reported and objectively-measured height, weight, and BMI were all strongly correlated (r = 0.96, 0.98, 0.96 respectively) for the sample overall, as well as across gender and race/ethnicity (Additional File 2).

The majority of subsamples in Table 1 had significant differences between their self-reported and objectively-measured height, weight, and BMI values. The extent of the misreporting varied by gender (Table 1). Cisgender men overreported their height statistically significantly more than cisgender women and TGD participants. There were not statistically significant differences in magnitude of misreporting by gender for weight or BMI.

Regression results revealed that reporting accuracy differed significantly across multiple participant characteristics (Table 2). Participants with obesity and overweight statistically significantly underreported their weight more than participants who were normal weight, and there were similar statistically significant misreported BMI. Participants with obesity also statistically significantly overreported their height more than participants with normal weight. Cisgender women and TGD participants overreported their height significantly less than cisgender men, and participants assigned female at birth reported their height more accurately than participants assigned male at birth. Cisgender women also underreported their BMI significantly less than cisgender men. Statistically significant differences by race/ethnicity were observed for Hispanic/Latine participants relative to white, AAPI, and multiracial participants on BMI misreporting, where the former were consistently underreporting their self-reported BMI. Also significant were AAPI participants, who overstated their BMI relative to Hispanic/Latine participants. Gay/lesbian/bisexual/pansexual/queer participants were more likely than heterosexual participants to underreport their height. No significant differences were found across age, household poverty status, and disordered eating behaviors.

**Table 2:** Associations between misreporting and participant characteristics

Characteristic	Height Misreporting Coefficient (95% CI)	Height *P* >|t|	Weight Misreporting Coefficient (95% CI)	Weight *P* >|t|	BMI Misreporting Coefficient (95% CI)	BMI *P* >|t|
Age	0.01 (−0.16 0.18)	0.874	−0.08 (−0.26, 0.09)	0.363	−0.03 (−0.11, 0.05)	0.459
Weight status (ref: normal weight)						
Underweight	0.13 (−0.82, 1.07)	0.793	1.05 (0.78, 2.02)	0.034*	0.41 (−0.02, 0.84)	0.063
Overweight	0.04 (−0.56, 0.64)	0.897	−0.92 (−1.53, −0.30)	0.004*	−0.43 (−0.70, −0.16)	0.002*
Obese	1.23 (0.50, 1.97)	0.001*	−1.81 (−2.57, −1.06)	0.000*	−1.30 (−1.64, −0.97)	0.000*
Gender (ref: cisgender men)						
Cisgender women	−1.41 (−1.95, −0.87)	0.000*	−0.22 (−0.78, 0.34)	0.439	0.30 (0.04, 0.55)	0.024*
Trans/genderqueer/nonbinary	−1.09 (−2.16, −0.02)	0.047*	0.32 (−0.79, 1.44)	0.571	0.45 (−0.06, 0.96)	0.081
Sex assigned at birth (ref: male)						
Female	−1.40 (−1.90, −0.91)	0.000*	−0.24 (−0.77, 0.30)	0.383	0.28 (0.04, 0.52)	0.024*
Sexual orientation (ref: heterosexual or straight)						
Gay/Lesbian/Bisexual/Pansexual/Queer	−0.63 (−1.22, −0.03)	0.040*	−0.36 (−0.97, 0.25)	0.241	0.02 (−0.26, 0.30)	0.893
Other sexual orientation	−0.59 (−1.69, 0.52)	0.300	0.70 (−0.43, 1.83)	0.224	0.47 (−0.06, 0.99)	0.080
Household poverty status (ref: At or below the poverty level)						
Above the poverty level	0.16 (−0.74, 1.06)	0.730	0.46 (−0.42, 1.35)	0.303	0.10 (−0.31, 0.50)	0.646
Missing income information	0.14 (−0.65, 0.94)	0.720	0.45 (−0.33, 1.22)	0.257	0.13 (−0.22, 0.49)	0.466
Disordered eating (ref: no)						
Yes	0.48 (−0.05, 1.01)	0.074	0.03 (−0.50, 0.56)	0.907	−0.16 (−0.41, 0.08)	0.189
Race/ethnicity (ref: white)						
Hispanic/Latine	0.39 (−0.57, 1.35)	0.428	−1.26 (−2.24, −0.28)	0.012*	−0.60 (−1.05, −0.15)	0.009*
AAPI	−0.16 (−1.13, 0.80)	0.738	−1.10 (−2.08, −0.12)	0.028*	−0.31 (−0.76, 0.14)	0.182
Other race/ethnicity	0.47 (−0.89, 1.83)	0.501	−1.04 (−2.42, 0.34)	0.140	−0.47 (−1.10, 0.16)	0.146
Multiracial	−0.06 (−1.26, 1.14)	0.920	−0.48 (−1.70, 0.74)	0.438	−0.12 (−0.68, 0.44)	0.672
Race/ethnicity (ref: Hispanic/Latine)						
White	−0.39 (−1.35, 0.57)	0.428	1.26 (0.28, 2.24)	0.012*	0.60 (0.15, 1.05)	0.009*
AAPI	−0.55 (−1.14, 0.04)	0.065	0.16 (−0.44, 0.76)	0.600	0.30 (0.02, 0.57)	0.035*
Other race/ethnicity	0.08 (−1.05, 1.20)	0.892	0.22 (−0.92, 1.36)	0.706	0.13 (−0.39, 0.66)	0.619
Multiracial	−0.45 (−1.38, 0.48)	0.340	0.77 (−0.16, 1.72)	0.106	0.48 (0.05, 0.91)	0.000*
Race/ethnicity (ref: AAPI)						
White	0.16 (−0.80, 1.13)	0.738	1.10 (0.11, 2.08)	0.028*	0.31 (−0.14, 0.76)	0.182
Hispanic/Latine	0.55 (−0.04, 1.14)	0.065	−0.16 (−0.76, 0.44)	0.600	−0.30 (−0.57, −0.02)	0.035*
Other race/ethnicity	0.63 (−0.50, 1.76)	0.272	0.06 (−1.08, 1.20)	0.918	−0.16 (−0.69, 0.36)	0.542
Multiracial	0.10 (−0.83, 1.03)	0.828	0.62 (−0.33, 1.56)	0.200	0.19 (−0.25, 0.62)	0.400
Race/ethnicity (ref: other race/ethnicity)						
White	−0.47 (−1.83, 0.89)	0.501	1.04 (−0.34, 2.42)	0.140	0.46 (−0.16, 1.10)	0.146
Hispanic/Latine	−0.08 (−1.20, 1.05)	0.892	−0.22 (−1.36, 0.92)	0.706	−0.13 (−0.66, 0.39)	0.619
AAPI	−0.63 (−1.76, 0.50)	0.272	−0.06 (−1.20, 1.08)	0.918	0.16 (−0.36, 0.69)	0.542
Multiracial	−0.53 (−1.86, 0.81)	0.437	−0.79 (−1.85, 0.27)	0.144	0.35 (−0.27, 0.97)	0.271
Race/ethnicity (ref: multiracial)						
White	0.06 (−1.14, 1.26)	0.920	0.48 (−0.74, 1.70)	0.438	0.12 (−0.44, 0.68)	0.672
Hispanic/Latine	0.45 (−0.48, 1.38)	0.340	−0.78 (−1.72, 0.16)	0.106	−0.48 (−0.91, −0.05)	0.029*
AAPI	−0.10 (−1.03, 0.83)	0.828	−0.62 (−1.56, 0.33)	0.200	−0.19 (−0.62, 0.25)	0.400
Other race/ethnicity	0.53 (−0.81, 1.86)	0.437	−0.56 (−1.91, 0.80)	0.420	−0.35 (−0.97, 0.27)	0.271

Overall, 15.4% of participants were classified into different BMI categories based on self-reported height and weight versus objectively-measured height and weight. Most misclassification events led to participants being classified into lower BMI categories than accurate (e.g., normal weight instead of overweight). Other than the 6.4% of participants with underweight who reported values which incorrectly classified them as normal weight, most participants with normal weight, overweight, and obesity who were classified into different categories depending on self-reported vs objectively-measured data had self-reports that placed them in one lower BMI category than their objective measurements designated. For example, 8.6% of the participants with normal weight would have been incorrectly classified based on self-reported data, and most of that group (69%) would have been categorized as underweight. Similarly, of the 28.6% of participants with overweight who would have been misclassified, the majority (90%) would have been categorized as normal weight, and of the 24.2% of participants with obesity who would have been misclassified, all of them would have been categorized as overweight.

**Fig. 1:**
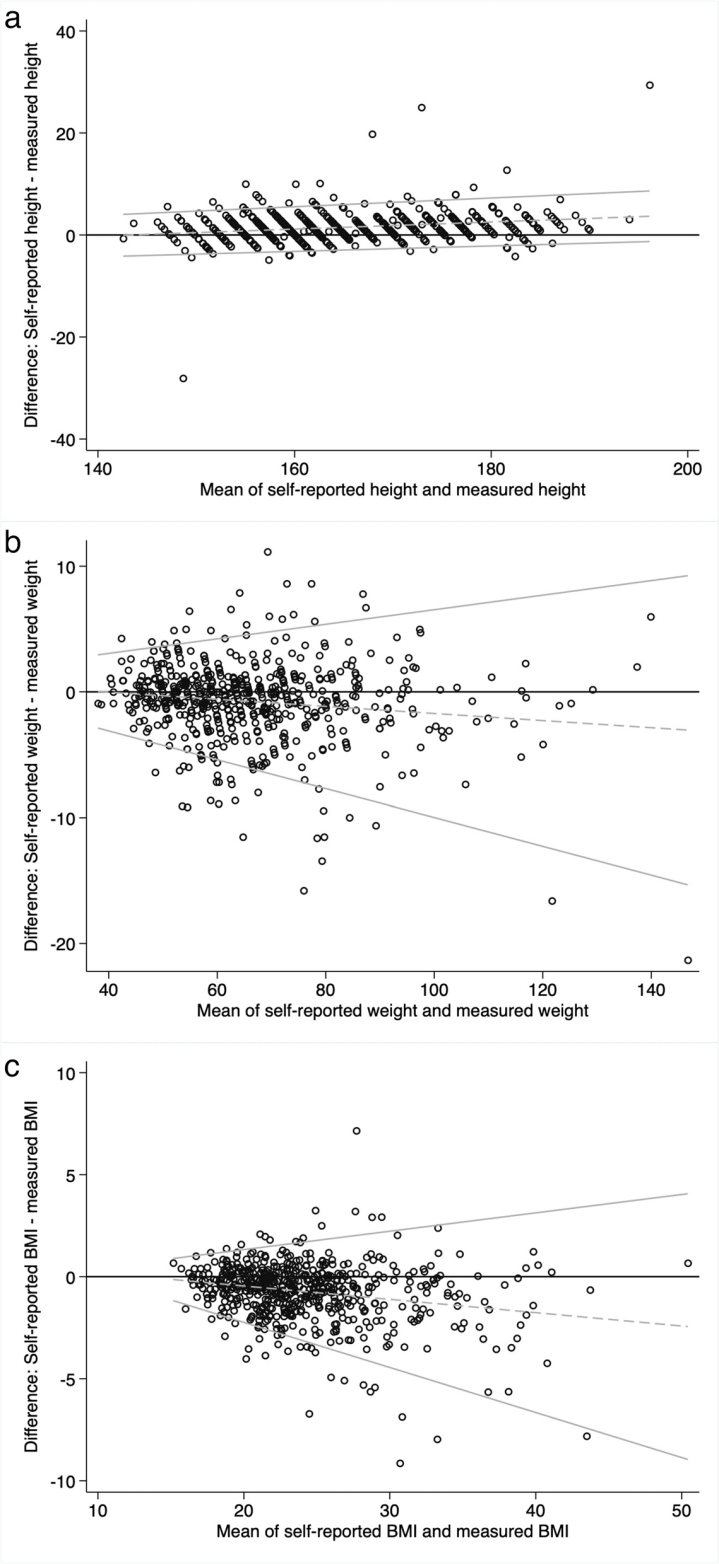
Bland–Altman plots for the overall sample. **a**. Bland–Altman plot comparing self-reported and measured height. **b**. Bland–Altman plot comparing self-reported and measured weight. **c**. Bland–Altman plot comparing self-reported and measured BMI

We constructed Bland–Altman plots (Figs. 1a, b and c) to assess variation in agreement between self-report and objective measures as the values of height, weight, and BMI increased. For height, the plot indicated a slight but statistically significant upward slope (0.07, *p* < 0.001), indicating more overreporting among taller participants. Downward slopes for weight (−0.03, *p* < 0.001) and BMI (−0.07, *p* < 0.001) and widening ranges at higher values for weight and BMI suggested a trend toward more underreporting and greater variability as weight and BMI increase.

### Sensitivity analyses

We conducted two sensitivity analyses. We first compared regression results by research site and found similar results across the two research sites (Additional File 3). Our second sensitivity analysis stratified those who had a gap of less than a week between completing the survey and health visit, and those with a gap of a week or more (Additional File 4). There was, on average, an 17-day delay between filling out the survey where participants self-reported their height and weight, and the health visit where it was objectively measured. During that period, participants could have fluctuated slightly in weight, which would have been perceived as inaccurate self-reporting in our analyses if they reported perfectly accurately at both times, which could add bias to the results. Some significant differences that were found in the overall sample were no longer significant when analyses were stratified for gaps in survey and health visit completion. Specifically, participants with overweight who had a gap of a week or more between survey and health visit completion did not underreport their weight or BMI significantly more than participants with normal weight who had the same gap. Additionally, Hispanic/Latine participants with gaps of a week or more did not underreport BMI significantly more than white participants. Women with gaps of both less than and equal to a week, or greater than a week, did not overreport their BMI compared to men after the sample was stratified by gap length.

## Discussion

In this study of diverse emerging adult college students, self-reported height and weight diverged from objective measurements, and the magnitude of some of these discrepancies varied by sex/gender and obesity status. Overall, BMI was on average underestimated by 0.75 kg/m^2^. Despite inaccuracies, nearly 85% of the overall sample was correctly classified into international adult BMI categories by self-report, and self-reported and objectively-measured height, weight, and BMI were all strongly correlated, supporting the utility of self-report as a practical, scalable method when resource constraints preclude objective measurement. Additionally, these findings may inform bias-correction approaches that account for participant characteristics for future application in studies of emerging adult populations.

The modest but statistically significant misreporting trends we found for height, weight, and BMI closely aligns with findings from previous studies with emerging adults [1, 8, 9]. However, magnitudes of misreporting varied. In comparison to other studies, the difference in self-reported and objectively-measured heights we found is consistently larger [1, 5, 8, 9, 11, 18], the difference in weights is generally smaller [1, 5, 8, 9, 18] (although one study found that participants, on average, self-reported higher weights than their actual weights [11]), and the difference in BMIs falls in between what has been observed previously [9, 18, 21]. Like other studies [2, 5, 11, 22], these misreporting trends were statistically significant. Notably, the variation in weight is similar to the magnitude of weight variation associated with menstrual cycles [27, 28] and daily weight fluctuations [29, 30].

When examining the alignment between self-reported and objective measurements across sociodemographic subgroups, we identified differences by sex/gender and obesity status. Cisgender women and TGD participants overreported their height significantly less than cisgender men, and those assigned female at birth were more accurate in reporting their height compared to those assigned male at birth. These findings align with previous research [1, 6, 18], but, to the best of our knowledge, this is the first study that has compared self-reported and objective measurement accuracy across gender with TGD participants. Additionally, our Bland–Altman plots found that taller participants overreported their height more than shorter participants. Given that men are, on average, taller than women, the plots corroborate our findings on men overreporting height more than women. Men with higher levels of stereotypically masculine gender role characteristics have been found to overreport their height to a greater extent than other men, which may also imply that the desirability that societal standards assign to taller men could explain men overreporting their height more than women and TGD participants [31].

Our regression models, Bland–Altman plots, and sensitivity analyses show that participants with obesity underreported their weight, and self-reported measurements that led to their BMI being underreported significantly more than participants with normal weight. Additionally, a much higher percentage of participants with underweight, overweight, and obesity were categorized into the incorrect weight group due to their inaccurate self-reported measurements than participants with normal weight. These findings corroborate prior research that participants with overweight and obesity are, in aggregate, less accurate in their weight reporting than people with normal weight [20, 32]. Participants with underweight were more likely to be incorrectly classified as having normal weight based on self-reported measurements, which supports previous findings that underweight people overreport their weight [14, 20]. A potential explanation for participants with overweight and obesity underestimating their weight to greater extents than participants with normal weight is that they are more consciously underreporting their weight to align with the general societal preference for smaller bodies [33, 34]. Similarly, people with underweight may overreport their weight to distance themselves from weight-related stigma, as evidenced by studies finding stigma towards people with anorexia nervosa [35, 36] and other eating disorders [37, 38].

Importantly, 24.7% of participants with overweight or obesity were incorrectly classified into a lower-weight BMI group based on their self-reported measurements. Misclassifying participants into different BMI groups can introduce bias into data, and lead to inaccurate conclusions, among other concerns. Multiple other studies on emerging adult college students’ self-report accuracy have found similar levels of BMI group misclassification [5, 11]. To mitigate the effects of BMI group misclassification, we recommend utilizing a continuous measure of BMI in future studies so that minor misreporting does not significantly impact data analysis.

### Strengths and limitations

This study has multiple important strengths. Compared to other studies, ours reports on the highest percentage of Hispanic/Latine and AAPI emerging adults, broadening knowledge beyond findings made from other largely white student samples [2, 19]. Similarly, to the best of our knowledge, we are the first to examine weight and height reporting accuracy among TGD emerging adult college students. We also analyze misreporting behaviors stratified by a wide variety of participant characteristics, many of which have never been analyzed in the existing literature (e.g., sexual orientation, poverty status, disordered eating).

The study also has some limitations. The 3E Study enrolls emerging adults attending two Hispanic-Serving Institutions of higher education, so the generalizability of our findings may not extend to all emerging adults. Also, because of the sample’s diversity, it is not representative of US college student demographics, or US emerging adults as a whole. The increased number of Hispanic/Latine, AAPI, and multiracial participants is reflective of projected demographic trends in the US [39]. We also note the limitation of delays between completing the survey and the health visit, which was addressed via sensitivity analyses. Additionally, participants’ clothing was not fully standardized, as they were only asked to remove shoes or heavy outer layers, which could have influenced cross-group variation. The weight of remaining clothing also could have contributed to the gap observed between measured and self-reported weights. Another limitation is that participants volunteered to join the study, which may have introduced volunteer bias. As a result of this bias, people who are more interested in health may be overrepresented in the sample, which may have made the sample less representative of the target population. Finally, we calculated mean differences for populations and population subgroups; extensive individual variation remains within groups that warrants future examination.

### Directions for future research and practice

Due to the sample size, we were not able to analyze TGD people stratified by race/ethnicity in this study. TGD people make up a growing portion of emerging adults [40], and future studies should prioritize recruiting gender diverse emerging adults to allow for the disaggregating of TGD categories (e.g., transgender men and transgender women), which could reveal further nuance. Additionally, our study adds to the limited literature on racial/ethnic differences in height/weight misreporting. Many studies on this topic have mainly consisted of white participants, so future research should continue to promote the inclusion of understudied racial/ethnic groups and provide more insight into racial/ethnic differences in misreported height and weight.

## Conclusions

Emerging adult college students are an increasingly diverse group that are at an inflection point for health, and are therefore critical for studying health trajectories. We add to current literature by identifying small levels of misreporting in a group that is more diverse in terms of gender, race/ethnicity, and sexual orientation than prior studies. Additionally, this is one of the first studies to report on data collected since the beginning of the COVID-19 pandemic, which increased body image concerns particularly for adolescent and emerging adult women [41, 42]. We found minor, but statistically significant inaccuracies in self-reported height and weight, including some differences by sociodemographic group, most notably by gender, in the extent of misreporting. Higher magnitudes of misreporting were also associated with higher participant values of height, weight, and BMI. Studies which compare height, weight, and BMI results across these groups should acknowledge that some differences could be due to varying levels of misreporting across these characteristics. However, the modest differences in self-reported and objectively-measured values do not suggest that it is imperative for studies to abandon self-report as a height, weight, and BMI data collection strategy when it is logistically beneficial.

While the magnitude of the errors for continuous measures (e.g., weight, height, BMI) were small, assigning participants into BMI categories amplified potential misclassification: 16% of participants were categorized into an incorrect BMI group due to their self-reported height and weight, which could potentially introduce bias into research findings.

Studies that rely on self-reported weight and height, rather than objective measurements, should be aware of small but systematic misreporting issues when interpreting their findings, and consider using continuous measures (e.g., BMI) rather than categorical measures (e.g., BMI category) to reduce potential misclassification bias.

## Supplementary Information

Additional file 1. Full study sample participant characteristics (including participants excluded from analytical sample). A table containing descriptive statistics of the full sample of the study, including the participants who were excluded from the analytical sample.

Additional file 2. A table containing Person’s correlations for self-reported and measured height, weight, and BMI for the sample divided by gender and race/ethnicity.

Additional file 3. Table 2 stratified by research site. A table detailing the same analysis as completed in Table 2, but with the sample divided by whether participants attend CSUEB or UCR.

Additional file 4. Table 2 stratified by gap between survey and health visit. A table detailing the same analysis as completed in Table 2, but with the sample divided by whether the gap between the participant completing the online survey and the health visit was less than a week or a week or more.

Additional file 5. Divided by gender and race/ethnicity: means for self-reported and measured height, weight, and BMI. A table containing mean self-reported and measured height, weight, and BMI divided by gender and race/ethnicity.

## Data Availability

The data supporting the conclusions of this article are not currently available but will be made available upon the conclusion of the grant period. Interested individuals can contact the author team with any reasonable data requests.

## Abbreviations

BMI: Body mass index
TGD: Transgender and gender-diverse
UCR: University of California, Riverside
CSUEB: California State University, East Bay
AAPI: Asian, Asian American, and/or Pacific Islander
